# An unusual case of incidental pancreatic neuroendocrine tumor presenting with main pancreatic duct cystic dilatation

**DOI:** 10.1093/omcr/omae104

**Published:** 2024-09-07

**Authors:** Samuel Essoun, Nii A Adu-Aryee, Henry E Obaka, Bernard Seshie, Dzifa Dey, Simon Naporo

**Affiliations:** Department of Surgery, Korle Bu Teaching Hospital, P.O. Box KB 77, Korle Bu, Accra, Ghana; Department of Surgery, University of Ghana Medical School, P.O. Box GP 4236, Accra, Ghana; Department of Surgery, Korle Bu Teaching Hospital, P.O. Box KB 77, Korle Bu, Accra, Ghana; Department of Surgery, Tema General Hospital, Hospital Road, Tema, Ghana; Department of Medicine and Therapeutics, University of Ghana Medical School, P.O. Box GP 4236, Accra, Ghana; Department of Pathology, Greater Accra Regional Hospital (Ridge Hospital), P.O. Box GP 473, Accra Ghana

**Keywords:** pancreatic tumor, neuroendocrine tumor, pancreatic cysts, pancreatic duct dilatation

## Abstract

Introduction: Pancreatic lesions have varied morphology and presentation making their diagnosis challenging. The lesions may be asymptomatic incidentalomas on abdominal imaging for other conditions, symptomatic producing specific hormone effects or causing local effects. Case: We report a 35-year-old woman with recurrent abdominal pain confirmed gastroesophageal reflux disease. Initial CT imaging reported findings of a pancreatic pseudocysts. A careful review of the imaging showed cystic dilatation of the main pancreatic duct mimicking a main pancreatic duct intra-ductal papillary mucinous neoplasm. At surgery, a small nodule palpated in the pancreatic head with sacculation in the body and tail. A histopathological review showed a pancreatic neuroendocrine tumour blocking the main pancreatic duct at the neck causing downstream dilatation and sacculation. This case highlights the difficulty of diagnosing small asymptomatic pancreatic tumours especially with limited range of imaging modalities while increasing awareness of these conditions to improve our ability to manage them effectively.

## Introduction

Pre-malignant cystic pancreatic lesions represent a clinicopathologic spectrum with the potential to progress into invasive cancer. Serous cysts are seen as being benign, whereas the mucinous cysts have malignant potential [1]. The reported prevalence of cystic pancreatic lesions is up to 49% in the general population, with an average of nearly 4 cysts per patient, and average size of cyst between 2 mm and 29 mm [2].

In turn, the commonest solid pancreatic lesions are pancreatic ductal adenocarcinoma, pancreatic neuroendocrine tumor (PanNET), solid pseudopapillary tumor and focal chronic pancreatitis [3]. PanNET, the second leading solid lesion may present a diagnostic challenge because of varying clinical presentation and features on pancreatic imaging. The lesions show atypical radiological features including solid, purely cystic, solid-cystic, calcified type, and diffuse forms [4]. We report a rare case of incidental PanNET co-existing and presenting as an asymptomatic cystic dilatation of the main pancreatic duct (PD).

## Case report

A 35-year-old woman with pre-existing systemic lupus erythematosus managed with regular hydroxyurea and corticosteroids and in stable state, reported to her physician with 2 months history of intermittent left hypochondriac pain. An abdominal ultrasonography reported a dilated PD (15.7 mm), and cyst at the tail measuring (26.2×2×22.9) mm, and an abdominal CT scan also reported similar findings, with both concluding on a diagnosis of pancreatic pseudocyst. The serum amylase was marginally elevated, however, serum lipase, IgG4, blood sugar and HbA1c were all normal. Her physician initially managed her empirically with antacids and analgesia and her symptoms resolved.

The symptoms recurred 5 months later, and a repeat abdominal CT scan reported a uniformly dilated PD measuring >28 mm with thinning of pancreatic parenchyma and a (5.6×5.5×5.1) cm cyst at the tail abutting the hilum of the spleen, and a diagnosis of distal PD stricture and a pseudocyst in the setting of chronic pancreatitis made. Surgical consult was sought and her evaluation with an esophago-gastroduodenoscopy revealed severe gastroesophageal reflux disease as the cause of her recurrent symptoms which resolved once appropriate medical treatment with proton pump inhibitors and prokinetics agents were commenced.

**Figure 1:**
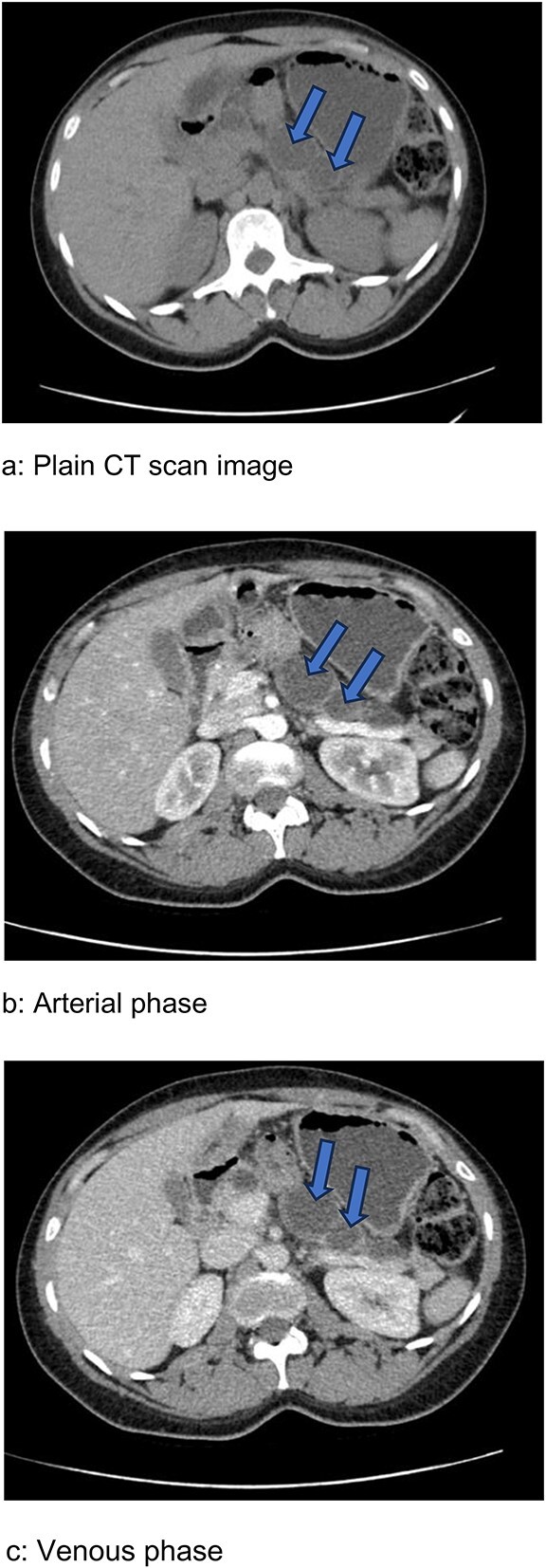
Abdominal CT scan images showing series of cystic dilatations (arrows) of the main pancreatic duct with thinned pancreatic tissue in the body and tail apparent in all three phases. (**a**) Plain image. (**b**) Arterial phase. (**c**) Venous phase.

Reviewing her abdominal CT images showed a series cystic dilatation of main PD from the neck to the tail with atrophic pancreatic tissue (Fig. 1a–c). The largest cyst located at the tail previously reported as a pseudocyst showed an interval change in size otherwise no other lesions were seen. The presumptive diagnosis was an asymptomatic main PD IPMN, the patient was counseled about the risk of malignant transformation and surgery recommended.

**Figure 2:**
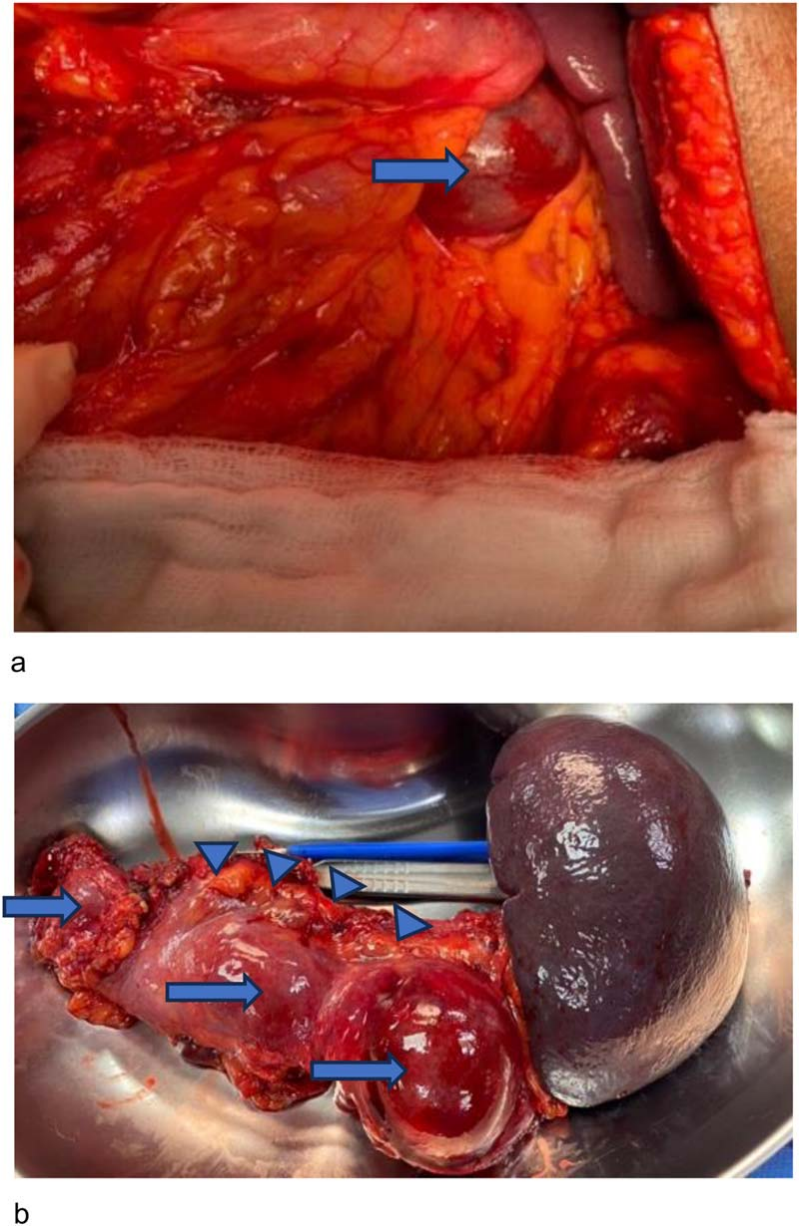
Intra-operative images showing cystic lesions shown with arrows. (**a**) Intraoperative image which shows the cystic swelling of the tail of the pancreas and the spleen with the arrrow showing the saccular lesion at the pancreatic tail. (**b**) Surgical resection specimen including the pancreatic body, tail and the spleen, showing a series of cystic swellings (arrows), a thin rim of pancreatic tissue (arrow heads) and the spleen.

Surgery was a distal pancreatectomy with a cuff of the pancreatic head resected. Findings of cystic dilatation of the pancreatic body and tail with very little pancreatic tissue (Fig. 2a and b), and a hard sub-centimeter nodule in the head, close to the neck was incorporated in the surgical resection.

Post-operative recovery was complicated by peri-pancreatic abscess which was drained percutaneously. She fully recovered and returned to work by 6 weeks after surgery. Subsequently, she has no complications and has returned to normal life and full function.

**Figure 3:**
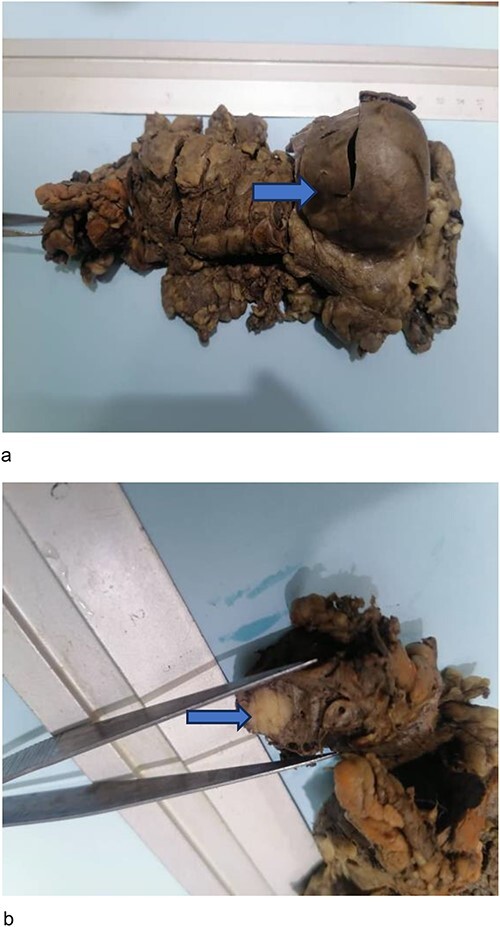
Gross pathology specimen. (**a**) Pathology specimen of pancreas fixed with formaldehyde without the spleen and arrow pointing to the cystic dilatation at the pancreatic tail. (**b**) Section shows well circumscribed pale looking tumor nodule (arrow) in the proximal segment of the resected surgical specimen (wedge of the pancreatic head).

Pathology confirmed 209 g pancreas with multiple sacculation expanding to a cyst 5.5 cm at the splenic hilum (Fig. 3a). The smooth inner lining of the sacculation contained dark brown fluid, communicated with the duct which continued towards the pancreatic resection margin, terminating in a solid firm cream to tan 11 mm nodule, with slightly irregular margin (Fig. 3b). The surrounding tissue was grey and fibrous except for adjacent region of recognizable pancreatic tissue towards the tail.

**Figure 4:**
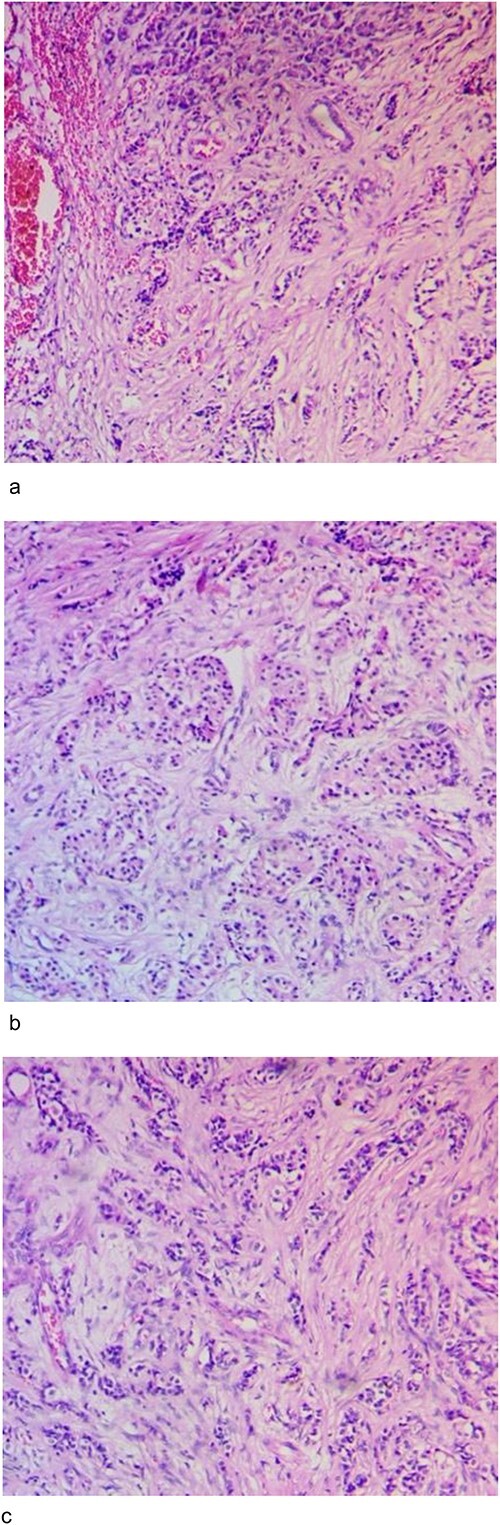
Histopathology images. (**a**) Histology shows trabecular and ductulo-glandular architecture (H&E 40X). (**b**) Histology showing trabecular and cord-like architecture composed of bland cells without mitosis or necrosis (H&E 40X). (**c**) Histology showing trabecular architecture composed of small round cells with hyperchromatic nuclear and pale scanty cytoplasm. (H&E 40X).

Microscopy confirmed the nodule to be a well differentiated tumour (bland lesional cells without obvious mitotic figures), without lymphovascular and perineural invasion, 0 out of 16 lymph nodes, and free resection margins (Fig. 4a–c).

**Figure 5:**
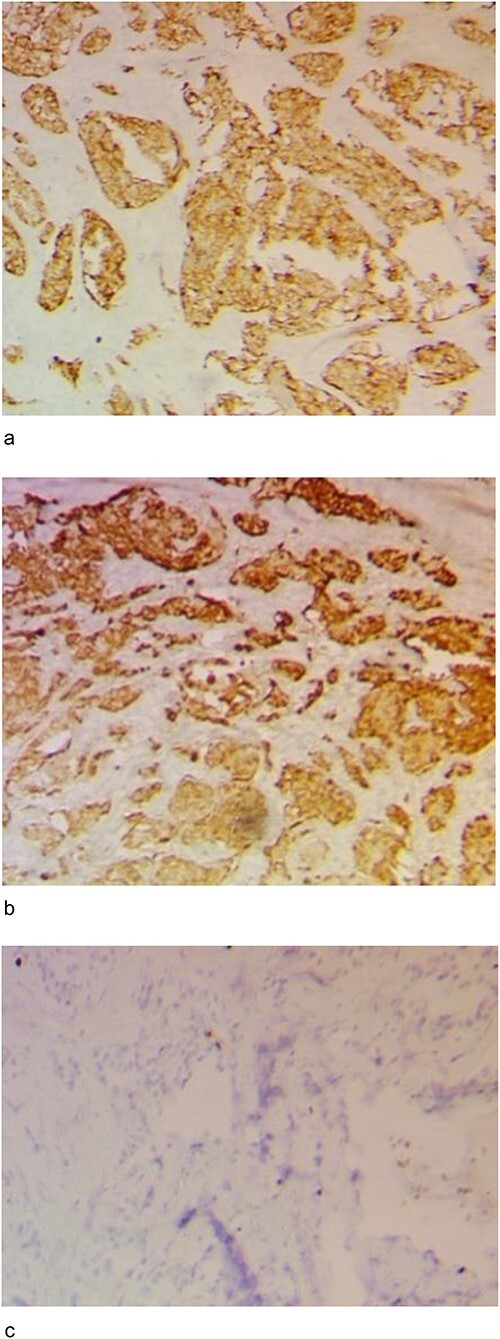
Immunohistochemistry images. (**a**) IHC Showing strong synaptophysin positivity (40X). (**b**) IHC Showing strong chromogranin positivity (40X). (**c**) IHC Showing very low Ki67 positivity (20X).

Immunohistochemical staining was positive for Synaptophysin (Fig. 5a) Chromogranin A (Fig. 5b) and Ki67 was <10% (Fig. 5c). These features confirmed an indolent PanNET within the pancreatic head with the cystic PD dilatation.

Post-surgery follow up has been uneventful, her blood sugars, HbA1c and serum chromogranin A has been normal 14 months after the surgery and no evidence of recurrence or pancreatic insufficiency. She continues to be monitored by her rheumatologist, primary physician and surgeons in a coordinated manner. Her monitoring included serial monthly weight check for the initial 6 months and at each clinic visit, 3 monthly fasting blood sugar and HbA1c, bi-annual serum chromogranin A and fecal elastase, and yearly abdominal CT scan which have all been normal so far.

## Discussion

This case highlights challenges in diagnosing pancreatic diseases. Our patient was thought to have main duct IPMN on abdominal imaging incidentally had a co-existing indolent PanNET not identified on CT imaging, although endoscopic ultrasound or other higher resolution imaging modalities not available in our practice may have helped. The narrative of a small (11 mm) indolent PanNET obstructing the main PD causing a series of cysts along the duct could also be true. Ishii et al. [5] reported a sub-centimetre PanNET blocking the main PD causing a uniform ductal dilatation without any sacculation or cysts as was in this case [5]. Larger malignant lesions can obstruct causing PD dilatation, as it has been recognized that, marked PD dilatation (≥3.5 mm) strongly suggests the presence of pancreatic disease [6]. Such is the rarity of this case where a small incidental PanNET causes a series of sacculation of the main PD, causing perhaps a rare type of retention cyst [1].

PanNETs are categorized as non-functional tumours secreting nonspecific peptides like chromogranin A, low quantity of hormones like pancreatic polypeptide and calcitonin [7] and malignant in 50% to 90%, have an indolent behavior and a better prognosis [8]. Functional tumours are uncommon and produce hormones and peptides including insulin, gastrin, vasoactive intestinal peptide (VIP), glucagon, somatostatin, and serotonin.

PanNET may be an incidentaloma on imaging or at surgery as was in this case or present due to activity of hormones secreted by the tumour or mass effect of large tumours. Its diagnosis can be made with conventional imaging techniques—ultrasonography (US), computed tomographic (CT) scan, or magnetic resonance imaging (MRI) which localizes and help stage the disease [8]. The detection rates with imaging depends on the size of lesion as lesions <10 mm are frequently missed. In this case, lesion was completely missed due in part to the size, and non-enhancing or being featureless on CT imaging. The absence of endoscopic ultrasound, advance functional and hybrid imaging using octreotide scintigraphy, single-photon emission computed tomography (SPECT) and positron emission tomography (PET)/CT and PET/MRI in our practice limited our abiility to further evaluate the pancreas.

Surgical resection offer the potential for cure in localized, non-infiltrative PanNETs without metastasis as was with our case [9].

In conclusion, we reported a rare case of a non-functional PanNET co-existing with large cystic dilatations of the main PD diagnosed only after surgery, highlighting the difficulty in diagnosing these lesions when they are small and not seen on conventional imaging.
